# A291 ANA-NEGATIVE AUTOIMMUNE HEPATITIS IN A 30-YEAR-OLD FEMALE: A DIAGNOSTIC CHALLENGE

**DOI:** 10.1093/jcag/gwad061.291

**Published:** 2024-02-14

**Authors:** A H Islam, R Mortuza, J Arab

**Affiliations:** London Health Sciences Centre, London, ON, Canada; London Health Sciences Centre, London, ON, Canada; London Health Sciences Centre, London, ON, Canada

## Abstract

**Background:**

Autoimmune Hepatitis (AIH) is a chronic liver condition arising from the immune system's attack on liver cells. Its diagnosis is typically supported by the presence of autoantibodies, notably ANA. However, cases have been identified where AIH occurs in the absence of these conventional serologic markers. Prior studies have reported that ANA-negative AIH patients showed more acute presentations and higher frequencies of histologic acute hepatitis compared to those with positive autoimmune serology. As such, ANA-negative subset of patients presents a diagnostic challenge but highlights the evolving understanding of autoimmune processes in liver diseases.

**Aims:**

To detail the clinical presentation, investigative measures, and treatment approach in a an otherwise healthy 30-year-old female diagnosed with ANA-negative autoimmune hepatitis.

**Methods:**

The diagnostic approach included clinical symptomatology, biochemical tests, imaging via abdominal ultrasound, and histopathological evidence from an ultrasound-guided liver biopsy

**Results:**

The patient presented initially with epigastric pain, jaundice, and pruritus over two weeks. Blood tests revealed acute hepatitis: ALT at 2723 U/L[JA1] , AST at 1428 U/L, and hyperbilirubinemia at 503 μmol/L, with GGT and ALP remaining normal. An abdominal ultrasound presented a typical liver morphology and biliary system. Initial autoimmune liver disease panels, including ANA, and viral hepatitis screenings, returned negative results. Furthermore, the patient's medication and drug history provided no additional clues for drug-induced aetiologies. For more conclusive diagnostics, an US-guided liver biopsy was performed during the admission revealing moderate acute hepatitis characterized by both interface hepatitis and lobular inflammation, prompting the suspicion for ANA-negative AIH. Initiating prednisone treatment (40 mg daily) based on clinical and biopsy evidence led to a significant reduction in liver enzymes and bilirubin. By the time of her discharge, her primary residual symptoms were mildly controlled jaundice and pruritus. She was transitioned to a tapering dose of prednisone, and Azathioprine was later added to her regimen. Subsequent close follow up revealed complete resolution of her symptoms. Notably, a 7-month follow-up unveiled a previously undetected AIH marker: ASMA with a titre of 1:80.

**Conclusions:**

ANA-negative AIH, as showcased in this case, emphasizes the importance of a thorough and iterative diagnostic approach. Traditional serological markers may not always be present, necessitating reliance on a combination of clinical presentations and histopathology. Regular follow-ups and periodic re-evaluations are essential. This approach is crucial for pinpointing the diagnosis, guiding effective therapeutic interventions, and ultimately ensuring positive outcomes for patients navigating complex autoimmune liver conditions.

**Funding Agencies:**

None

